# Immune System Dysfunction and Inflammation in Hemodialysis Patients: Two Sides of the Same Coin

**DOI:** 10.3390/jcm11133759

**Published:** 2022-06-28

**Authors:** Susanna Campo, Antonio Lacquaniti, Domenico Trombetta, Antonella Smeriglio, Paolo Monardo

**Affiliations:** 1 Nephrology and Dialysis Unit, Department of Internal Medicine, Papardo Hospital, 98158 Messina, Italy; susannacampo79@gmail.com (S.C.); ant.lacq@gmail.com (A.L.); 2 Department of Chemical, Biological, Pharmaceutical and Environmental Sciences, University of Messina, 98122 Messina, Italy; dtrombetta@unime.it (D.T.); asmeriglio@unime.it (A.S.)

**Keywords:** hemodialysis, immune system dysfunction, inflammation

## Abstract

Biocompatibility in hemodialysis (HD) has considerably improved in recent decades, but remains an open issue to be solved, appearing essential to reduce systemic inflammation and enhance patients’ clinical outcomes. Clotting prevention, reduction in complement and leukocyte activation, and improvement of antioxidant effect represent the main goals. This review aims to analyze the different pathways involved in HD patients, leading to immune system dysfunction and inflammation. In particular, we mostly review the evidence about thrombogenicity, which probably represents the most important characteristic of bio-incompatibility. Platelet activation is one of the first steps occurring in HD patients, determining several events causing chronic sub-clinical inflammation and immune dysfunction involvement. Moreover, oxidative stress processes, resulting from a loss of balance between pro-oxidant factors and antioxidant mechanisms, have been described, highlighting the link with inflammation. We updated both innate and acquired immune system dysfunctions and their close link with uremic toxins occurring in HD patients, with several consequences leading to increased mortality. The elucidation of the role of immune dysfunction and inflammation in HD patients would enhance not only the understanding of disease physiopathology, but also has the potential to provide new insights into the development of therapeutic strategies.

## 1. Introduction

Ten percent of the world population suffers from chronic kidney diseases, with 2.6 million people undergoing hemodialysis (HD), which will reach about 5.4 million in 2030 [1]. During the past 50 years, HD techniques have progressively improved, with a consequent strong impact on patients’ outcomes and quality of life [2].

Nevertheless, these patients are still chronically exposed to systemic stress related both to hemodynamic and non-hemodynamic factors, with increased risk for cardiovascular, neoplastic, and infection diseases [3,4].

In addition to established cardiovascular risk factors frequently observed in HD patients, such as dyslipidemia, blood hypertension, or diabetes mellitus, the additional activation of the immune system, involving both innate and adaptive responses, contribute to maintaining a condition of chronic systemic inflammation [5].

The concept of “inflammaging” identifies a “persistent, low-grade, sterile, non-resolving inflammatory state, associated with the senescence of the immune system” [6]. Thus, HD “per se” contributes to the morbidity and mortality of these patients inducing a systemic stress condition, resulting from hemodynamic management (weight loss, ultrafiltration), treatment schedules, solute fluxes, electrolytic shifts, and interaction between blood and the extracorporeal circuit [7].

The term “biocompatibility” was firstly used in 1970, although its first official definition was presented in 1986 when it was described as “the ability of a material to perform with an appropriate host response in a specific application” [8,9]. 

In the following years, this definition was modulated, pointing out the “interaction” between devices and human tissues [10], and taking into account the concepts of “bioactivity” [11,12]. 

During each HD session, the patient’s flowing blood leaves the physiological protection of the endothelial cells in the vessels and comes into contact with the extracorporeal circuit, with consequent physical and chemical stimulations.

These inflammatory stimuli and oxidative stresses start with the venipuncture of the arteriovenous fistula, and are then maintained by the interactions between blood and the extracorporeal HD circuit membrane with consequences for coagulation and the immune system [13,14,15]. 

In recent decades, the improvement of membrane biocompatibility has been one of the main targets of bioengineering applied to the HD field [16,17,18].

The so-called “first-use syndrome” was related to the old cellulose-based cuprophane membranes, due to the immunoreactivity of the free-hydroxyl groups, associated with a transient leukopenia and acute pulmonary dysfunction with leukocyte sequestration into the lungs [19].

Then, the free-hydroxyl groups were gradually substituted in modified cellulose-based membranes, developing synthetic and less-immunogenic membranes.

The mechanisms leading to the incompatibility reaction are still partially unclear, involving platelets and the coagulation system, the immune system, and the complement pathway [20,21]. 

## 2. Platelets and Coagulation System

Thrombogenicity is probably the most important characteristic of the bio-incompatibility of artificial material, and the activation of platelets is one of the first steps occurring in HD patients, determining several events causing chronic sub-clinical inflammation and immune dysfunction. 

Platelets bind to the filter membrane surface due to a blood–biomaterial interaction. Some adsorbed proteins, such as collagen, fibronectin, and fibrinogen, bind to glycoprotein IIb/IIIa receptors, promoting platelet adhesion. Then, platelets release their granular content and they aggregate, leading to the complex process of the thrombus formation.

At the same time, the absorption processes act as a trigger for the intrinsic pathway of coagulation, activating factor XII, prekallikrein, and kininogen. The consequence is a reaction cascade-activating factors X and II, leading to thrombin generation, acting on fibrinogen to form an insoluble fibrin “clot” [22]. 

While the cleavage of kininogen generates bradykinin, stimulating the release of pro-inflammatory cytokines [23,24], the coagulative pathway is amplified by the activation of the factor IX, which binds the activated factor VIII and factor X, leading to the production of thrombin and then to fibrin generation and platelet activation [25,26]. 

Thrombin, per se, triggers numerous pro-inflammatory effects, inducing cytokines and chemokine synthesis and the expression of adhesion molecules from endothelial cells, causing endothelium permeability and vascular remodeling [27]. 

During HD therapies, multiple stimuli amplify and trigger these processes, apart from the dialysis membrane contact. The needle used for venipuncture, blood tubing, trauma caused by blood pumps, temperature of the dialyzer, and the bubble trap chamber all are sources of significant activation of both coagulation and platelets [28,29]. 

Moreover, the visible clots that could appear in the HD circuit represent only the final process of the activated coagulation pathway. 

Prothrombin fragment analyses, thrombin–antithrombin III complex, and d-dimer evaluation can assess the “pre-clotting” stages, as well as platelet-to-lymphocyte ratio, obtained by dividing the absolute platelet count by the absolute lymphocyte count, which could represent a novel marker of inflammation in HD patients [30,31,32].

The quantitative analysis of the platelet does not highlight the real issue. Their count typically decreases in the first 30 min of dialysis and then stabilizes or returns to the pre-dialysis state at the end of dialysis treatment [33]. 

The qualitative dysfunction of the platelets is linked to their atypical activation, mainly affected by the type of dialysis membrane. In particular, some reports analyzed the incidence of thrombocytopenia observed in patients treated with polysulfone membranes [34,35]. 

The fall in platelet count observed with polysulfone membranes has been attributed to platelet activation, rather than complement activation, as revealed in the recent decades, during cuprophane membrane use [36]. Conversely, synthetic membranes based on polysulfone or polyethersulfone, but blended with polyvinylpyrrolidone as a hydrophilic agent, had an excellent biocompatibility profile, reducing protein fouling and platelet adsorption [37,38]. Furthermore, the use of heparin does not block the coagulation and platelet activation steps, unlike the more effective anticoagulant citrate. More specifically, regional citrate anticoagulation has been shown in in vitro and in vivo studies to reduce platelet and leukocyte activation, as well as complement activation, in a dose-dependent manner [39].

In a recent study, Orsag tested the effect of variable doses of citrate on biocompatibility parameters in HD patients, observing that 3 mmol/L of citrate abolished platelet activation, with no changes in the clotting score of the HD circuit [40].

## 3. Innate Immune System

### 3.1. Complement

Complement is one of the major components of the innate immune system and bridges the adaptive response of the body to abnormal stimuli, as well as being induced by hemodialysis, with consequent inflammation and pro-coagulant effects [41,42].

All the three pathways of the complement activation (classical pathway (CP), lectin pathway (LP), and alternative pathway (AP)) are involved; it is known that they all converge on C3 convertase, an enzymatic complex that generates C3a and C3b factors through C3 cleavage, and they can be activated by different triggers, such as acetylated compounds, carbohydrate structures, proteins adsorbed on biomaterials, and immunoglobulin G [43]. 

During the first 10–15 min of the HD session, C3a levels increased, indicating C3 activation, and subsequently C5a and C5b levels also raised, with an increase of up to 70% of soluble C5b9 levels and plasmatic C3d/C3 ratios during a single treatment of HD [44]. 

However, this complement activation effect is active in the early stages of HD and gradually decreases during long-term dialysis, as revealed by the negative correlation between C3 levels and dialysis duration [20].

The first studies, conducted on cellulose-based HD membranes, revealed the activation of the alternative pathway of the complement system. However, the lectin and classical pathways are also activated by HD, respectively, by the binding of mannose-binding-lectin and ficolin-2 (for LP) and properdine and/or C3b (for CP) to the dialysis membrane [45,46]. Moreover, polysulfone membranes can adsorb some complement inhibitors, such as factor H and clusterin, significantly reducing their circulating amount, further complementing activation [45,47]. 

Conversely, the use of medium cut-off filters decreased the levels of many complement components, including C4B, when compared to polyamix membranes [48].

Interventions targeting the complement system could improve biocompatibility, dialysis efficacy, and long-term outcomes. As observed for the platelet activation, citrate inhibits complement activity through calcium chelation in the HD circuit [49]. 

Complement inhibitors could represent other attractive therapeutic options to reduce complement activation and inflammation. Poppelaars observed that the addition of C1-inhibitor to an ex vivo HD model significantly reduced the complement activation and the induction of pro-inflammatory cytokines, such as TNF-α, IL-6, and von Willebrand factor [20]. In an ex vivo model of HD, Kourtzelis used compstatin to block complement activation at the C3 level, improving the biocompatibility of hemodialysis membranes [50]. 

A modified polysulfone membrane with a direct thrombin inhibitor, Argatroban, was chemically grafted to enhance the hydrophilicity and induced protein adsorption, coagulation, and platelet and complement activation [51]. 

### 3.2. Neutrophils and Monocyte Macrophages

The interaction between blood and biomaterials during the HD session also stimulates the cellular components of the innate immune system, mostly neutrophils and monocytes macrophages. Their recruitment and the subsequent release of pro-inflammatory cytokines contribute to maintaining the pro-inflammatory status and thus increasing cardiovascular risk in HD patients [52].

Many studies analyzed the changes in leukocyte count induced by dialysis sessions, although the results have sometimes been inconstant. Fukushi examined peripheral leukocytes and neutrophils counts in HD patients treated with polysulfone membranes, revealing a decrease in neutrophils number at the end of the HD session and a significant increase in apoptosis-positive cells among neutrophils and monocytes, but not among lymphocytes. The activation of the complement system and the increased apoptotic cell levels mainly caused this transient leukopenia [53].

Bieber confirmed this datum, measuring high levels of neutrophil activation and death markers, such as calprotectin, peroxidase activity, and neutrophil extracellular traps (NETs), in HD patients treated with polysulfone membranes [54]. 

Moreover, Koga assessed the effects of five different polysulfone membranes on blood cells in vitro, showing considerable differences in platelet adhesion and reactive oxygen species production by neutrophils. The number of adherent platelets and reactive oxygen species production increased with the amount of fibrinogen adsorbed on the membranes, suggesting that the use of dialyzers with lower fibrinogen adsorption may reduce cell activation, microvascular inflammation, and oxidative stress during HD [55]. 

Whereas neutrophil numbers could transitory change, qualitative alterations characterized monocytes, with modifications of phenotype and functions, contributing to their dysfunction. 

Monocytes are highly plastic cells able to modify their initial phenotype when facing environmental modifications, such as those in HD patients, with important consequences on their ability to interact with vascular structures, causing chronic inflammation [56].

Monocytes can be classified into three subpopulations (Mo1, Mo2, and Mo3) based on the expression of different surface markers. Mo1 monocytes show a “classical” pattern expressing lipopolysaccharide (CD14), but not the Immunoglobulin Fc Segment Receptor (CD16), while Mo2 and Mo3 monocytes express both CD14 and CD16 [57].

Mo2 monocytes act as antigen-presenting cells showing an “inflammatory pattern”, since they produce inflammatory factors, such as tumor growth factor (TGF)-β1.

Dialyzed patients have abnormally high proportions of intermediate (CD14^++^/CD16^+^) Mo2 and Mo3 monocytes, with pro-inflammatory and atherogenic features, and a strong ability to attach to endothelial cells, thus contributing to endothelial damage, and are consequently associated with atherosclerotic disease and cardiovascular events [58,59].

Liakopoulos analyzed the surface-marker profile of monocytes from HD patients treated with polysulfone membranes, confirming a skewed distribution of pro-inflammatory Mo2 and Mo3 monocytes. Moreover, behind this atypical pattern, monocyte had phenotype alterations inducing a functional impairment after a single dialysis session. In particular, the authors described a significant reduction in the chemokine receptor CX3CR1 expression in all monocyte subpopulations, impairing their adhesion to the endothelium during hemodialytic treatment. In vitro analyses confirmed the significant decrease in CX3CRI surface expression on monocytes after incubation with foreign uremic serum, suggesting a uremia-related impaired immune response. Finally, supporting the previous observations, HD patients’ monocytes showed an impaired response to lipopolysaccharide stimulation, mirroring the immune dysfunction [60]. 

The potential role of different dialysis techniques in modulating monocytes’ phenotype and function has been investigated with conflicting results. Some authors reported a reduction in the Mo2 population in patients treated with online hemodiafiltration, when compared to standard HD, without differences between pre-, mixed, or post-dilution [61,62]. 

However, a prospective trial based on hemodialysis with high cut-off membranes or surface modification of cuprophane dialyzers with the antioxidant vitamin E failed to reduce pre-dialysis levels of inflammatory monocytes and related markers, notwithstanding high amounts of pro-inflammatory cytokines cleared [63,64]. 

These conflicting data could be related to the differences between the membranes analyzed, with different cellular activation signals. Measuring monocytes before and after a dialysis session can be influenced by the dialysis-induced sequestration of cells, which may considerably change the cell population distribution in peripheral blood. However, the more biocompatible membranes remove more Mo2 and Mo3 cell populations from circulation during dialysis than Mo1 cells, as a measure of dialyzer membrane biocompatibility [65]. 

The Mo3 cells reach a nadir at about 15–30 min of a dialysis session and return to pre-dialysis levels until the end of treatment at 4–5 h [66].

Impairment and activation are two sides of the same coin involving the immune natural cells in HD patients, with reduced defense mechanisms, such as phagocytic capabilities or impairment of antigen presentation function, and, on the other side, increased synthesis of inflammatory cytokines.

## 4. Acquired Immune System

### T and B Cells

The dysfunction of the adaptive immune response characterizes HD patients with negative implications for morbidity and mortality. Many studies described a reduced number and functional alterations of naïve T cells, Th2, and regulatory T cells [67], while highly differentiated memory T cells increase [68]; these cells show a pro-inflammatory phenotype destabilizing atherosclerotic plaques and enhancing the inflammatory state [69].

T-cell lymphopenia observed in HD patients seems to be due to impaired thymic output, increased apoptosis, and reduced proliferation [70,71].

Starting from these assumptions, the HD treatment “per se” can contribute to adaptive immune system dysfunction [72]. 

Borges reported that HD procedure contributes to the development of T-cell lymphopenia, at least in part, by apoptosis induction, with negative effects on CD4^+^ T cells also mediated by recombinant erythropoietin (rhuEPO) therapy, often administered in these patients [73]. Moreover, an increased CD4^+^/CD8^+^ T-cell ratio, after a single hemodialysis session [74], and a weakened response of CD4^+^ T cells to mitogen-mediated stimulation, have been revealed [75]. 

All these conditions, characterized by a loss of telomere length, reduced expression of activation antigens, and impaired proliferative capacity, could be related to a stress-induced premature senescence (SIPS) process, involving changes in the function and morphology of cells in response to the chronic inflammatory process [76].

CD4^+^ T lymphocytes of HD patients are characterized by impaired proliferation parameters, such as a reduced number of cell divisions, a longer period required by these cells to enter the first (G1) phase of the first cell cycle, and a decreased percentage of cells able to divide [75].

Adaptive immune response dysregulation in HD patients also involves B lymphocytes. As for T cells, an increase in high differentiated forms and a reduction in naïve cells has been described [77]. 

One of the possible explanations could be found in the increased levels of soluble CD40 in patients undergoing hemodialysis. CD40 and its ligand (CD40L) regulate several cellular functions, including T- and B-cell activation, but their interaction is antagonized by the soluble form of CD40 [78]. 

## 5. Inflammation and Oxidative Stress

HD patients are affected by an inflammatory state with multifactorial pathogenesis, resulting in increased morbidity and mortality [79].

Inflammation is due to HD-related factors, such as dialysate quality, membrane compatibility, dialytic age, and vascular access, by oxidative stress, infections, and patient-related factors (comorbidities) [5].

Oxidative stress results from a loss of balance between pro-oxidant factors and antioxidant mechanisms. In HD patients, higher plasmatic levels of pro-inflammatory cytokines, such as tumor necrosis factor α (TNFα) and interleukin 6 (IL-6), have been reported, and intracellular levels of reactive oxygen species (ROS) are also increased in this patient population [80,81]. 

Similar results were obtained in the HD children population, whose IL-6 levels were significantly higher when compared to subjects affected by stage 5 CKD and healthy children. Moreover, IL-6 levels rose with increased time of dialysis [82].

Plasma levels of endothelin-1 (ET-1), a potent coronary vasoconstrictor, are also increased and they are associated with adverse clinical events in HD patients. ET-1, acting as a mediator for leukocyte recruitment, induces the expression of leukocyte adhesion molecules and the synthesis of inflammatory mediators, enhancing neutrophil adhesion to endothelial cells [83].

In a recent study, Hirayama evaluated the effects of hemodialysis with high-flux polysulfone membranes on multiple ROS using electron spin resonance-based methods. They concluded that ROS scavenging activities deteriorate after a single HD session, suggesting an uncontrolled production of these radicals during HD [84]. 

This “pro-oxidant” environment results in the formation of oxidized lipids or advanced oxidation protein products (AOPPs) and the expression of pro-inflammatory cytokines and recruitment of pro-inflammatory cells mainly through Nuclear Factor Kappa B (NF-κB) stimulation [85].

The presence of bacterial DNA in the dialysate can induce C-reactive protein (CRP) and IL6 production, further increasing oxidative stress. In HD patients, a decreased intracellular pH value, due to a lower concentration of pre-dialysis plasma bicarbonate, contributes to the creation of a pro-oxidative environment [86]. 

Many other pro-oxidative factors, such as anemia and iron administration, should be taken into account. On the other hand, chronic kidney disease is characterized by a progressive impairment of the antioxidant systems [87].

Vascular access also plays a role in inducing HD patients’ inflammatory state. Previous studies have reported an increased mortality ratio in patients with central venous catheters compared to those with native AV fistula, due to the worst dialysis quality, increased infection incidence, and inflammatory state [15,88]. 

In a recent study, the authors compared inflammation and micro-inflammation parameters in patients with AV fistula and with central venous catheters: the latter showed a higher degree of inflammation independently from catheter infections, while the vascular access was not associated with higher mortality rates [89]. Finally, many studies have reported a positive correlation between oxidative stress and mortality in HD patients [90].

## 6. Uremic Toxin Involvement

The accumulation of uremic toxins increased leukocyte activity and inflammation. The link between the immune system and cardiovascular damage is based on endothelial damage, representing the starting point of the cascade of events leading to cardiovascular disease. Uremic endothelial cells may be involved in the activation of innate immunity, but they may also be damaged by this immune activation [91]. In particular, several uremic toxins increase the expression of adhesion molecules, such as ICAM-1, VCAM-1, and E-selectin, and inflammatory and chemoattractant factors, such as TNF-α and MCP-1 in endothelial cells, as well as the activation and adhesion of leukocytes to the endothelium [92].

Uremic toxins chronically activated Toll-like receptors (TLRs), involved in innate immunity, inducing the production of pro-inflammatory DAMP (danger signal-associated molecular pattern) levels, such as HMGB1, amplifying the inflammatory milieu. The vicious circle is closed by other DAMP receptors signaling activation, behind TLR, such as NLR-inflammasome-activated caspase-1 and other pro-inflammatory cytokines, which increase uremic toxins levels and inhibit CD4^+^ regulatory T cells [93].

Uremic peptides compromised coagulation and fibrinolysis mechanisms, inducing pro-coagulant activity in endothelial cells by increasing tissue factor expression and consequently the factor Xa formation [94]. Furthermore, uremic toxins directly contribute to cardiovascular complications by reducing NO synthesis in endothelial cells, impairing endothelial cell proliferation, amplifying pro-inflammatory effects, and altering immune processes [95,96].

The immune system is critical in maintaining homeostasis with the resident microbiota, and on the other hand, resident microbes influence the immune response [97].

Significant endotoxemia, related to a gastrointestinal stasis due to excess fluid and reduced clearance of uremic toxins, permanently stimulated the immune system and altered permeability and loss of intestinal epithelial barrier integrity [98].

This is another link between inflammation and immune dysfunction, mediated by the kidney–gut crosstalk, with a pivotal role played by the intestinal barrier function and bacteria. Under inflammatory conditions in HD patients, uremic toxins of bacterial origin alter the intestinal barrier function, and in the circulation, those uremic toxins stimulate immune cells [99]. 

This altered gut barrier facilitates systemic translocation of gut bacterial DNA and products of bacterial protein catabolism, well-known as microbiota-derived uremic toxins, such as indoxyl sulfate, p-cresyl sulfate, and indole-3 acetic acid, detectable in the blood of HD patients [100].

In addition to the traditional and these microbiota-derived uremic toxins, several new predictors of cardiovascular events have recently been recognized, such as lipoprotein-associated phospholipase A2, a serine lipase produced by activated monocytes, which induces the chemotaxis of leucocytes into the lipid core of the atherosclerotic plaque, transforming it into a necrotic and instable core [101]. 

In this context, it is pivotal to reduce the burden of traditional uremic and microbiota-related toxins, such as p-cresyl and indoxyl sulfate, in HD patients.

Several uremic compounds are identified in serum and plasma samples from CKD patients, and their classification occurs according to their behavior during dialysis [102].

**Figure 1:**
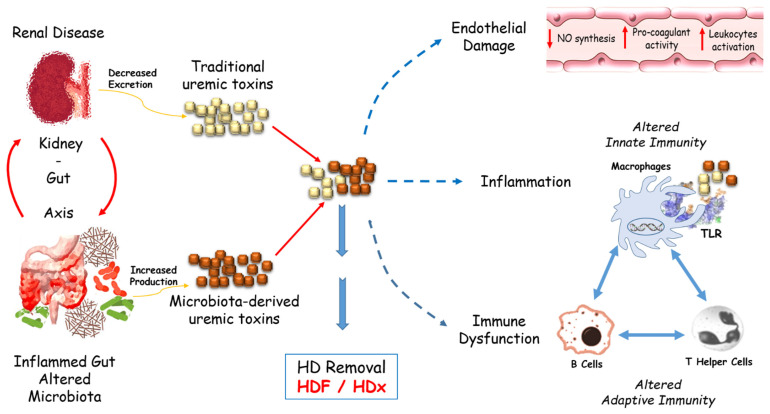
Crosstalk between uremic gut and kidney, leading to traditional and microbiota-derived uremic toxins. Abbreviations: HDF: hemodiafiltration; HDx: expanded hemodialysis; NO: nitric oxide; TLR: Toll-like receptor.

Hemodiafiltration and medium cut-off (MCO) dialyzers applied to standard hemodialysis, defined as “expanded hemodialysis” (HDx), improved the elimination of middle- to large-sized molecular toxins [103,104], see Figure 1.

## 7. Future Perspectives

Although biocompatibility in hemodialysis has considerably improved during the recent decades, it remains an open issue for researchers. 

Several approaches have been adopted to ameliorate the anti-fouling, hemocompatibility, and antibacterial activity of dialysis biomaterial. The main goals to achieve are clotting prevention, reduction in complement and leukocyte activation, and improvement of antioxidant effects. 

The dialysis circuit geometry plays a key role, so bioengineers and designers aimed to realize more compact blood cassettes and shorter tubing systems to minimize the blood–air interface. A second key point is reducing bacterial contamination and consequent endotoxin release using endotoxin-retaining filters, endotoxin-retaining membranes, and improving sterilization techniques. 

However, the main research field concerns improving the biocompatibility of dialysis membranes. Researchers are trying to reach this ambitious goal by different approaches, such as chemical modifications of the biomaterial surface, thus reducing or changing its reactivity; modifications of the surface charge; and attachment or coating of surfaces with biofunctional entities, such as anticoagulants, antiplatelet agents, and antioxidants. 

Vitamin E antioxidant effects are well known and it has been used in the last two decades to treat HD-related inflammation. Moreover, many authors documented the anti-inflammatory and antioxidative effects of vitamin-E-coated membranes. 

Yang reported that the long-term use of vitamin-E-coated dialyzers has enhanced ROS scavenger activity [105]. 

Sepe compared the effects of low-flux HD bicarbonate, low-flux HD bicarbonate with vitamin-E-coated membranes, and hemodiafiltration on nitric oxide formation and Indoleamine 2,3-dioxygenase-1 (IDO1) activity. They observed that chronic HD patients treated with more biocompatible vitamin-E-loaded hemofilter showed reduced IDO1 activity and NO formation when compared to chronic HD patients treated with polysulfone membrane dialyzers [106].

In the recent years, several novel surface modification techniques have been attempted. 

In 2016, Bensaadi reported that the addition of polyvinylpyrrolidone (PVP) and poly-ethylene-glycol (PEG) within cellulose triacetate hybrid dialysis dialyzers enhanced membrane morphology and reduced the adsorption/adhesion of macromolecules [107].

In a study published in 2021, Venkatesh and his group described the fabrication and testing of a PES (polyetheresulfone) dialysis membrane surface-grafted with zwitterion (so with a neutral overall charge) TiO2 nanofiber brushes. The zwitterion fibers showed high anti-fouling activity and very high biocompatibility in terms of protein absorption and platelet adhesion [108].

In the same year, Meyer tested the safety of a novel dialysis membrane realized by mixing polysulfone, polyvinylpyrrolidone, and a fluorinated polyurethane surface-modifying macromolecule (SMM) named Endexo. The authors hypothesized that the incorporation of Endexo within dialyzer fibers may reduce the adhesion and activation of blood proteins and platelets providing a passive and low-energy surface [109] (Table 1). 

## 8. Conclusions

HD patients are at increased risk of cardiovascular disease, neoplastic diseases, and infections. Three times a week they undergo a life-sustaining therapy representing a repetitive stress condition because of its intrinsic “unphysiological” nature. Thus, hemodialysis “per se” contributes to the morbidity and mortality of these patients.

Hence, improving dialysis material biocompatibility appears essential to reduce systemic inflammation and enhance patients’ clinical outcomes. 

Promising innovations are arising, especially in terms of surface grafting methods for the design of bioactive material interfaces. 

## Figures and Tables

**Table 1:** Immuno-inflammatory dysfunction and future perspectives in HD patients: from theory to clinical practice.

Pathway	Mechanisms	Potential Biomarkers	Potential Therapy
*Complement* *System*	Activation of the complement response, inducing pro-coagulant state, releasing inflammatory mediators from immune cells with vascular endothelial damage and atherosclerosis Reduction in expression of complement inhibitors due to dialyzer absorption (CFH, an inhibitor of C3 convertase and C3b, ficolin-2) [45,46,47]	Ficolin-2 reduction C3a increase C5a increase C5b increase Soluble C5b9 increase C3d/C3 ratio increase	HDF/MCO/ HDx [48] Anticoagulant citrate [49] Polysulfone grafted with argatroban [51]
*Innate* *Immunity*	Decrease in neutrophils due to activation and apoptosis [53] Monocyte (CD14^++^/CD16^+^) Mo2 and Mo3 phenotypes attach to endothelial cells, contributing to inflammation and endothelial damage	HMGB1 Calprotectin NETs [54] Monocyte subpopulations	Online HDF [61,62]
*Acquired* *Immunity*	Reduced and not-functional naïve T cells, Th2 and regulatory T cells, showing a pro-inflammatory phenotype [67] Altered B lymphocytes with increased high differentiated forms and a reduction in naïve cells [77]	T-cell lymphopenia increased CD4^+^/CD8^+^ Increased soluble CD40	No data
*Coagulation System and Platelet* *Activity*	Pro-thrombotic status mediated by activated intrinsic and extrinsic pathways leading to pro-inflammatory effects and endothelial cell damage Platelet dysfunction with atypical activation	D-dimer, β-TG TAT	Anticoagulant citrate [39,40] Polyvinylpyrrolidone [37,38]
*Oxidative* *Stress*	Loss of balance between pro-oxidant factors and antioxidant mechanisms	ROS dosage [80,81] Oxidized lipids AOPP	Vitamin-E-coated filter [106]
*Uremic* *Toxin*	Endothelial dysfunction Inflammation Immune dysfunction	FLC, microbiota-derived uremic toxins, lipoprotein-associated phospholipase A2	HDF/MCO/HDx [103,104]
**Future Perspectives**			
**Materials**	**Mechanisms**	**Potential Biomarkers**	**Effects**
Vitamin-E-coated filter	Enhanced ROS scavenger activity	ROS NO IDO1 [106]	Anti-inflammatory antioxidative
PVP and PEG filters	Enhanced membrane morphology and reduced adsorption/adhesion of macromolecules [107]	No data	Anti-inflammatory antithrombotic
PES filter grafted with zwitterion	Anti-fouling activity and high biocompatibility in terms of protein absorption and platelet adhesion [108]	No data	Anti-inflammatory antithrombotic
Endexo	Reduced adhesion and activation of blood proteins and platelets [109]	No data	Anti-inflammatory antithrombotic

Abbreviations: AOPPs: advanced oxidation protein products; β-TG: β-thromboglobulin; CHF: complement factor H; Endexo: polysulfone, PVP: fluorinated polyurethane surface-modifying macromolecule; FLC: free light chains; HDF: hemodiafiltration; HDx; expanded hemodialysis; HMGB1: high-mobility group box-1; IDO1: indoleamine 2,3-dioxygenase-1; MCO: medium cut-off; NETs: neutrophil extracellular traps; NO: nitric oxide; PEG: poly-ethylene-glycol; PES: poly-etheresulfone; PVP: polyvinylpyrrolidone; ROS: reactive oxygen species; TAT: thrombin–antithrombin complex.
